# Allergy education and training for physicians

**DOI:** 10.1016/j.waojou.2021.100589

**Published:** 2021-11-10

**Authors:** Sally Barker, Lydia Daniels, Yoon-Seok Chang, Tinatin Chikovani, Audrey DunnGalvin, Jennifer D. Gerdts, Roy Gerth Van Wijk, Trevor Gibbs, Rosalaura V. Villarreal Gonzalez, Rosa I. Guzman-Avilan, Heather Hanna, Elham Hossny, Anastasia Kolotilina, José Antonio Ortega Martell, Punchama Pacharn, Cindy E. de Lira Quezada, Elopy Sibanda, David Stukus, Elizabeth Huiwen Tham, Carina Venter, Sandra N. Gonzalez-Diaz, Michael E. Levin, Bryan Martin, John O. Warner, Daniel Munblit

**Affiliations:** a Faculty of Medicine, Imperial College London, London, United Kingdom; b Department of Internal Medicine, Seoul National University Bundang Hospital, Seoul National University College of Medicine, Seongnam, Republic of Korea; c Department of Immunology, Tbilisi State Medical University, Tbilisi, Georgia; d Applied Psychology and Paediatrics and Child Health, University College Cork, Cork, Ireland; e Department of Paediatrics and Paediatric Infectious Diseases, Institute of Child's Health, Sechenov First Moscow State Medical University (Sechenov University), Moscow, Russian Federation; f Food Allergy Canada, Toronto, Ontario, Canada; g Section of Allergology, Department of Internal Medicine, Erasmus Medical Center, Rotterdam, the Netherlands; h Association for Medical Education in Europe (AMEE), Dundee, Scotland, UK; i Independant Consultant in Primary Care and Medical Education; j Regional Center of Allergy and Clinical Immunology, University Hospital “Dr. Jose Eleuterio Gonzalez”, Faculty of Medicine, Autonomous University of Nuevo León, Monterrey, Mexico; k Imperial College London, London, United Kingdom; l Pediatric Allergy and Immunology Unit, Children's Hospital, Ain Shams University, Cairo, Egypt; m Health Sciences Center. Autonomous University of Hidalgo State, Mexico; n Department of Pediatrics, Siriraj Hospital, Mahidol University, Bangkok, Thailand; o Asthma, Allergy and Immune Dysfunction Clinic, Twin Palms Medical Centre, Harare, Zimbabwe; p Department of Pathology, Medical School, National University of Science and Technology, Bulawayo, Zimbabwe; q Division of Allergy and Immunology, Department of Pediatrics, Nationwide Children's Hospital, Columbus, OH; r Department of Paediatrics, Yong Loo Lin School of Medicine, National University of Singapore, Singapore, Singapore; s Khoo Teck Puat-National University Children's Medical Institute, National University Hospital, National University Health System, Singapore, Singapore; t Human Potential Translational Research Programme, Yong Loo Lin School of Medicine, National University of Singapore, Singapore; u Section of Allergy and Immunology, University of Colorado Denver School of Medicine, Children's Hospital Colorado, Colorado, USA; v Division of Paediatric Allergy, Department of Paediatrics, University of Cape Town, Cape Town, South Africa; w Division of Allergy and Immunology, Department of Otolaryngology, The Ohio State University, Columbus, OH, USA; x Inflammation, Repair and Development Section, National Heart and Lung Institute, Faculty of Medicine, Imperial College London, London, United Kingdom; y Department of Infectious Disease, Faculty of Medicine, Imperial College London, London, United Kingdom; z Solov'ev Research and Clinical Center for Neuropsychiatry, Moscow, Russian Federation

**Keywords:** Allergy, Allergy education, Allergy training, Multidisciplinary team, Competence

## Abstract

The increasing prevalence of allergic diseases has placed a significant burden on global healthcare and society as whole. This has necessitated a rapid development of “allergy” as a specialist area. However, as allergy is so common and, for most, relatively easy to diagnose and control, all clinicians need to have basic knowledge and competence  to manage  mild disease and recognize when referral is required. The allergology specialty has not yet been recognized in many countries and even where allergy is fully recognized as a specialty, the approach to training in allergy differs significantly.

In the light of recent developments in allergy diagnosis and management, there is an urgent need to harmonize core competences for physicians, as well as the standardization of core principles for medical education and post-graduate training in allergy. All physicians and allied health professionals must appreciate the multidisciplinary team (MDT) approach to allergy, which is key to achieving the highest standards in holistic care. Due to worldwide variation in resources and personnel, some MDT roles will need to be absorbed by the treating physician or other healthcare professionals. We draw particular attention to the role of psychological input for all allergy patients, dietetic input in the case of food allergy and patient education to support all patients in the supported self-management of their condition on a daily basis. A strong appreciation of these multidisciplinary aspects will help physicians provide quality patient-centered care.

We consider that harmonization of allergy components within undergraduate curricula is crucial to ensure all physicians develop the appropriate allergy-related knowledge and skills, particularly in light of inconsistencies seen in the primary care management of allergy. This review from the World Allergy Organization (WAO) Education and Training Committee also outlines allergy-related competences required of physicians working with allergic patients and provides recommendations to promote harmonization of allergy training and practice worldwide.

## Background

Over the past few decades, the prevalence of allergic and auto-immune diseases has been increasing worldwide.^1^ Many hypotheses have been proposed to explain this proliferation of non-communicable diseases, but hitherto there are no proven strategies to prevent them. Despite improved understanding of mechanisms and management of established allergic disease, there remain many unmet needs within allergy care.

The chronicity of allergic diseases, their increasing prevalence and consequent impact on quality of life and healthcare services, make it a significant, albeit elusive, target for national and international public health interventions. World Allergy Organization (WAO) published the  WAO  White  Book on  Allergy in 2010 and an updated version in 2013^2^ which highlighted that although the burden of allergic diseases has increased over the last 40 years, we have not seen a corresponding increase in service provision and training to address this. It was estimated that approximately 20% of the global population is affected by allergic diseases.^3^ This disconnect is echoed in a consensus statement released by the International Collaboration in Allergy, Asthma, and Immunology (iCAALL) which draws attention to shortcomings in allergy training,^4^ and in the European Academy of Allergy and Clinical Immunology (EAACI) advocacy manifesto on “Tackling the  Allergy Crisis in Europe”.^5^

Allergy is often associated with affluent countries.^6^ However, the prevalence of allergic diseases is increasing in developing countries as populations move from rural to urban environments.^7^ A disproportionate health burden is carried by patients in developing countries who often face suboptimal environmental conditions and impeded access to effective medical care, compounded by a lack of allergy specialists.^8^ It is also noted that not first but second generation migrant populations to an allergy-prevalent country acquire the increased risk of allergy and asthma.^9^ The findings imply that prolonged, generational exposure to an altered, allergy inducing environment increases the predisposition to allergy manifestations. The WAO White Book on Allergy emphasizes the importance of considering the cost-effectiveness of our approaches to allergy. It is thought that improving undergraduate and postgraduate training and access to skilled allergy specialists should reduce costs by minimizing misdiagnoses or under-appreciation of allergy severity, thereby improving health outcomes.^2^

Existing literature demonstrates global heterogeneity in allergy education and training,^10^, ^11^, ^12^ which may be compromising patient care. This mandates the optimization of allergy education and training at all levels. WAO is well positioned to provide guidance and recommendations for standardization of training and care to improve patient outcomes regardless of geographical location. This review aims to look at how allergy features in undergraduate education, how allergy competences are defined, how specialist allergology training pathways vary, and how skills from other disciplines are needed within the provision of specialist care. For each of these aspects, we make recommendations which promote the harmonization of allergy training and practice worldwide. We anticipate that this review will play a key role in working towards ensuring physicians have access to appropriate education and training in allergy to facilitate high-quality, patient-centered allergy care.

## Allergy and clinical immunology as a part of undergraduate medical curricula

There is a need to ensure medical graduates have the appropriate scientific foundation in allergy and immunology upon which they can build clinical competence as they begin practicing. A comprehensive list of undergraduate curriculum components has previously been outlined by Potter et al on behalf of WAO.^13^ However, such recommendations have not been uniformly incorporated into undergraduate curricula. It has been proposed that lack of allergy representation in undergraduate teaching contributes to both the low numbers of physicians recruited to the allergy specialty and the overall inadequacies seen in allergy care.^14^^,^^15^

Several authors have carried out local analyses to map allergy teaching within undergraduate curricula. Shehata et al^14^ carried out a systematic analysis to map where allergy teaching lies within the planned curriculum of the University of Edinburgh Medical School. They highlighted gaps in taught content, including angioedema, insect and food allergies, interpreting the main diagnostic tests, managing co-morbidities, and indications for specialist referral. A second study^16^ surveyed those responsible for delivering teaching to find out whether these gaps were remedied in the "delivered curriculum", and confirmed a deficit in allergy-related topics.

Reid et al^11^ sought to assess allergy undergraduate teaching across multiple UK institutions. Teaching consultants linked to The British Society for Allergy & Clinical Immunology (BSACI) were surveyed about allergy teaching in their respective undergraduate curricula. Student representatives from each university were also surveyed to corroborate the consultants’ reports. Marked inconsistency in allergy teaching was shown across the United Kingdom, with insufficient allergy teaching, clinical exposure to allergy, and allergy-specific clinical skills training noted in the majority of institutions. However, it is possible that allergy is covered in other course components, such as primary care or pediatrics. In the United States, for example, undergraduate curricula are often “organ-based”, in which many diseases treated by the allergist are discussed within modules on dermatology, otolaryngology, and pulmonary medicine.^17^

An international survey on undergraduate allergy education is needed to establish whether this heterogeneity in allergy medical education exists worldwide. It is likely that further action is required to promote harmonization. Reid et al^11^ propose the standardization of allergy teaching in the undergraduate curriculum, including a guaranteed clinical placement in allergy for senior medical students. This would be a logical allocation of time and resources in developed countries with higher disease prevalence, but this may not be possible on a worldwide scale.

**Fig. 1:**
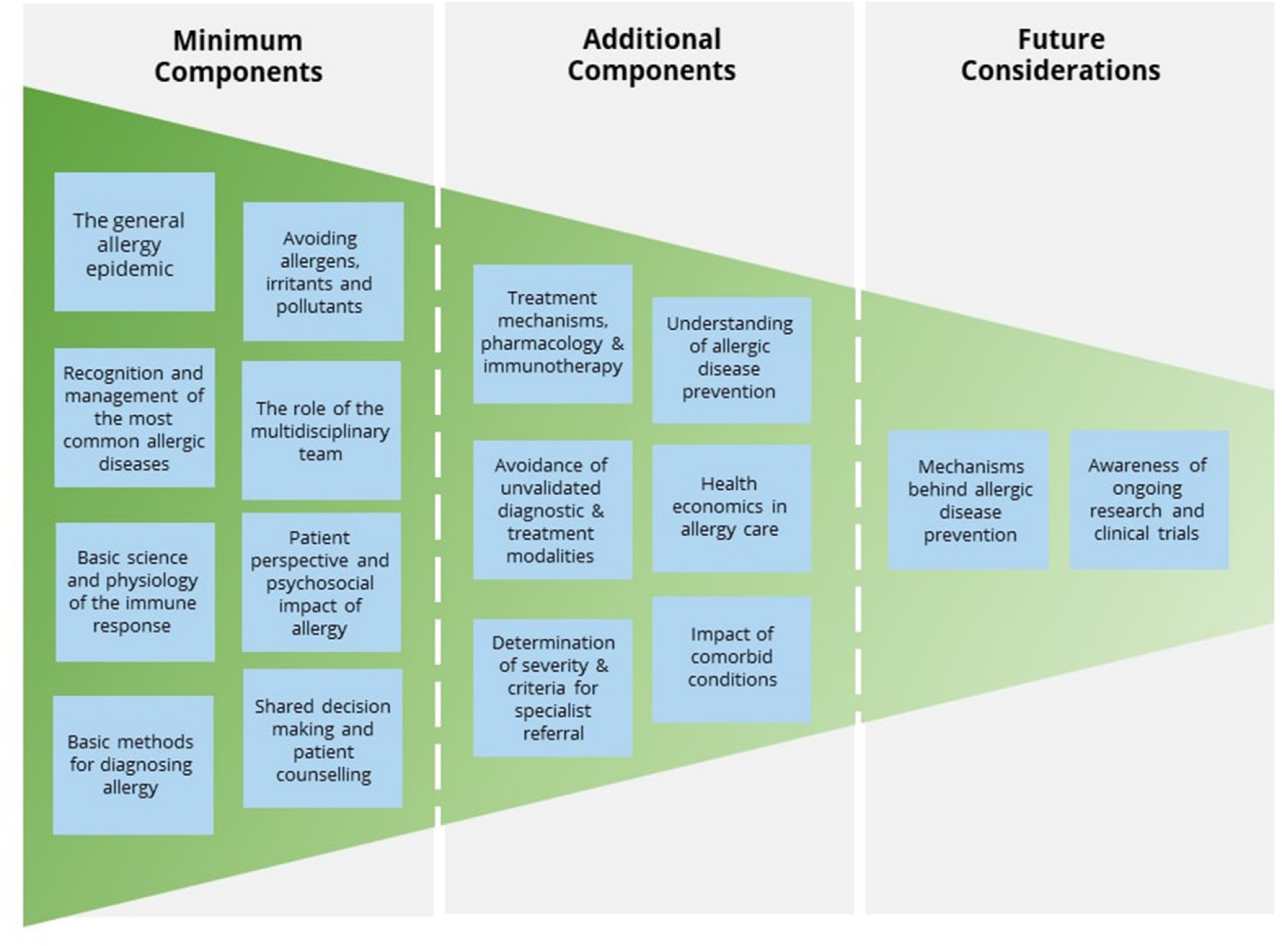
Recommended components for the undergraduate curriculum, stratified into “minimum components”, “additional components” and “future considerations”. ∗MDT = multidisciplinary team.

With all this is mind, we have developed an updated tiered system of allergy components, or topics, for integrating into the undergraduate curriculum, mapped out in Fig. 1. The curricula must have inbuilt flexibility to adapt to the emerging developments in allergy care.^18^ These components can be formulated into learning objectives by the individual medical school.

Improved understanding of immunological mechanisms of allergy has translated into improved diagnostic and treatment options available in daily clinical practice,^19^ hence we have classed basic science and physiology of the immune response as a "minimum" essential component. The concepts of innate and adaptive immunity are generic for virtually all medical disciplines, and allergy provides excellent examples. Understanding family history, the environmental and psycho-social influences on allergic disease is fundamental to practice and therefore provides an excellent example of complete history taking. We have also included awareness of the psychosocial consequences of allergic disease as a "minimum" component, to ensure graduates are equipped to provide holistic allergy care. "Additional components" and "Future considerations" include topics which educators could cover to stretch allergy awareness further, for example, ongoing research and trials which are likely to have breakthrough impact on allergy care.

With ever increasing demands to include more topics in the undergraduate curriculum, as allergy and other non-communicable diseases increase commensurate with reductions in infectious diseases, we view "allergy" as a prime example of a long-term, evolving condition which demands a multidisciplinary, holistic approach. Allergy as a taught subject would also allow students to explore how demographic changes affect disease susceptibility and health economics.

We consider it likely that adoption of these recommendations into worldwide undergraduate education will substantially improve standardization of knowledge, skills, and practice and improve patient care. This "grass-roots" approach^20^ may also improve recruitment to the allergy specialty, empower students to advocate for recognition of the specialty, and lead to further local development of allergy infrastructure in the future.

## Physician competences in allergy

Moving forward from undergraduate education, we look at how allergy competences for physicians have previously been outlined and propose a new model of competence requirements.

In 2008, WAO produced a report which outlines physician competences which are necessary for the adequate care of patients with allergic disease.^21^ The recommendations are broadly stratified according to the physician's level of training. The first level is made up of primary care physicians, general internists, and other non-allergy system specialists, with these competences ideally taught within the undergraduate medical curriculum.^13^ The second level represents care delivered by physicians who receive allergy training as a part of other specialist training, such as those in pediatric, dermatological, rheumatological, and respiratory specialties. The third level consists of specialists in allergology, who are expected to have a full breath of competence, including the management of multi-system allergic diseases and an in-depth understanding of complex psychosocial needs.

**Fig. 2:**
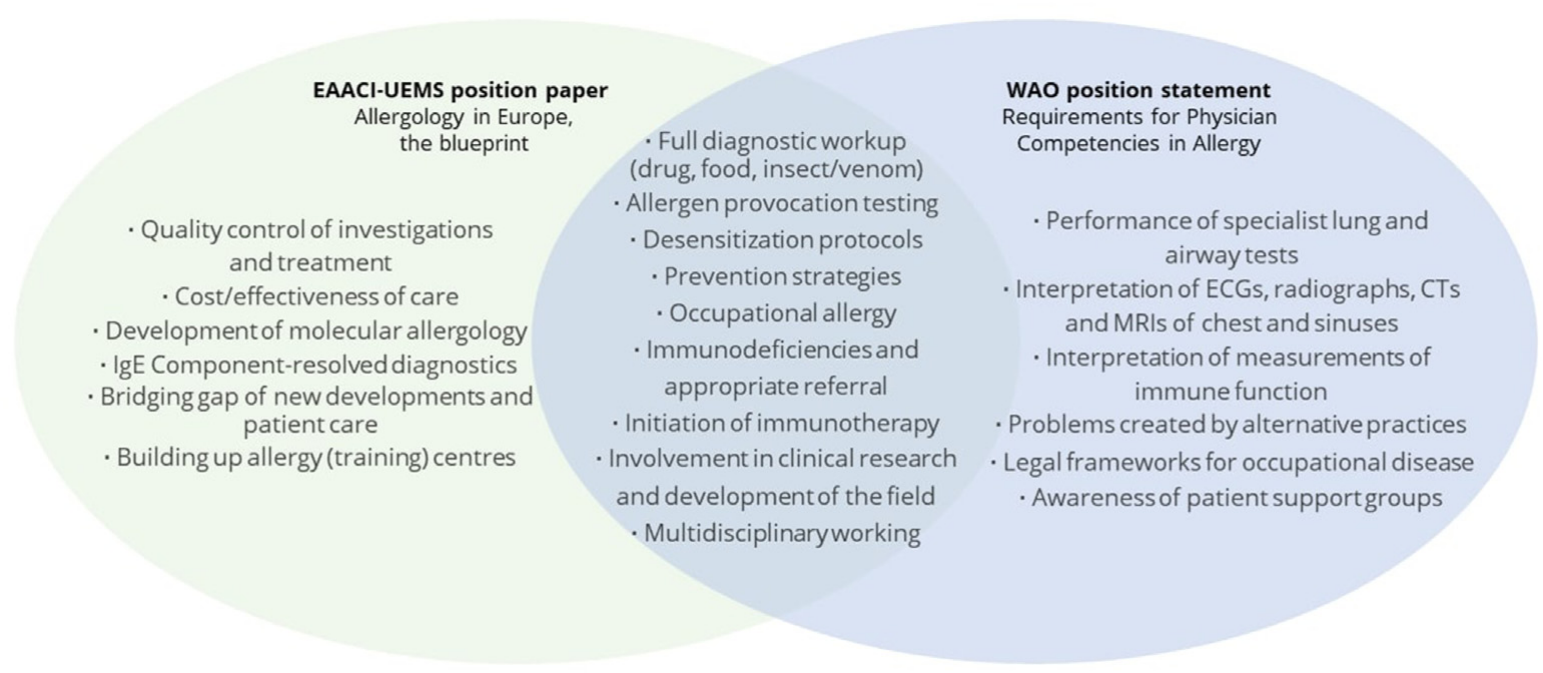
**Variation in prior competence recommendations for allergy specialists (EAACI and WAO)**.^21^^,^^22^ The overlapping area indicates where the WAO and EAACI recommendations agree. The lists either side are only noted by either WAO or EAACI-UEMS and represent recommendations unique to each paper.

Alongside this, other organizations such as the EAACI,^22^ have released position statements detailing their recommendations for specialist competences, which can act as a blueprint for allergy training programs. Notwithstanding the common recommendations seen across the board, there is significant variation in the skills and knowledge expected of allergy specialists. We demonstrate the similarities and differences between the WAO and EAACI position papers in Fig. 2.

The application of the WAO 2008 framework depends upon the healthcare structures and availability of specialists in each respective region. In an attempt to mitigate this, WAO provided guidance on adaptations that can be made according to local requirements, resources, and infrastructure.^23^

We wish to emphasize that there is a flexibility in how competence in allergy can be obtained, and that this may not strictly align with the “levels” which have been previously described. Therefore, we have reimagined the framework of competences into "core competences" required of any graduate physician, "additional competences" which could be relevant for specialists whose work involves a significant allergy component or primary care physicians who seek additional accreditation in allergy, and "specialist competences" for those qualifying in the allergy specialty. We consider that this is a better fit for the current landscape of allergy care, with the majority of care delivered in primary care settings, and other cases managed in partnership with other specialists such as pediatricians, pulmonologists, dermatologists, or rheumatologists. All physicians who encounter allergic patients should possess the "core competences", built upon a scientific grounding in allergy and immunology ideally taught in medical school as previously outlined in the "core components" in Fig. 1.

We echo the prior recommendation of WAO that physicians involved in allergy care should practice within a “cooperative network”,^21^ wherein physicians are aware of the limits of their own competences and refer to an allergy specialist or other relevant colleagues when necessary, and consider that our updated framework would also support this. It is important that all treating physicians have the opportunity to obtain "additional competences" in allergy alongside their existing training, particularly in regions where allergy specialization is not yet established. The "additional competences" could also align with training in the allergy subspecialty offered in some countries, proposed by EAACI as a “stop-over” to developing a full allergy specialty.^24^ In all cases, physicians should be aware of major position statements and guidelines regarding allergic disease, and seek up-to-date information from national and international professional bodies, associated peer-reviewed journals, and conferences.

Comprehensive knowledge of diagnostic tests is essential for physicians with "additional competences". Examples include skin prick testing and its applicability to the local context, titrated and fresh food testing, approaches to drug allergy diagnosis, routine tests for chronic urticaria, and specific inhalation provocation tests. An in-depth understanding of challenge procedures (oral food challenges, drug challenges, etc) is also expected. An awareness of allergen avoidance is expected as a "core competence", however more detailed knowledge of allergens and associated avoidance strategies is required by physicians with "additional competences", such as knowledge around allergen epitope specificity, immunogenicity, cross reactivity, local prevalence, seasonality, suitability as testing reagents, vaccines, and adjuvants. In the case of food allergens, they should understand the role of additives in adverse reactions to foods, the safety of vaccines for allergic patients and issues around the regulation of food labelling. Physicians with "additional competences" should also have appropriate knowledge of drug allergens, including the risk of cross-reactivity and suitable drug alternatives.^25^

**Fig. 3:**
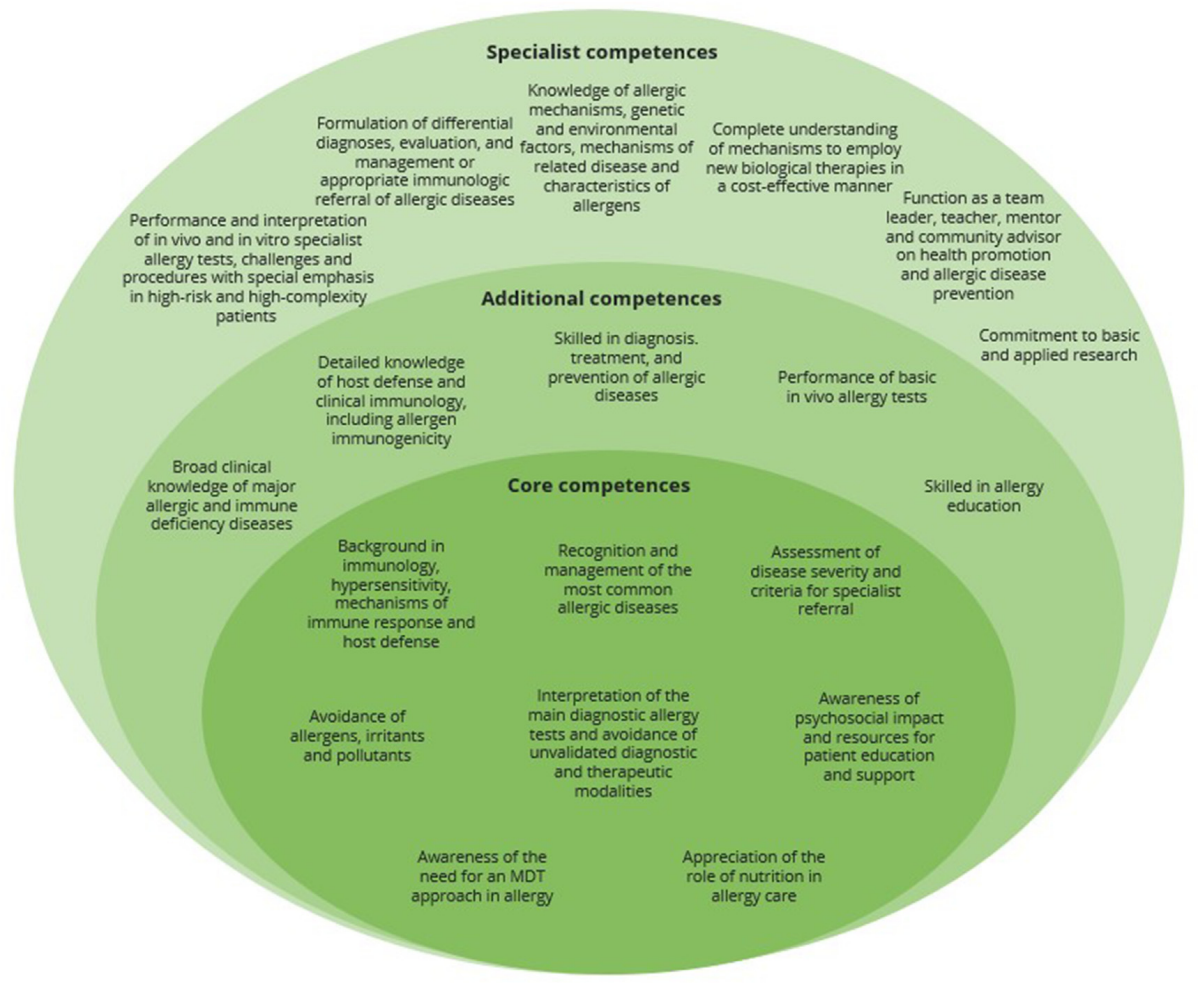
Proposed recommendations for competences in allergy stratified into “core”, “additional” and “specialist” competences. ∗MDT = multidisciplinary team.

Advanced investigation and tailored treatment of specific allergic diseases is considered to fall under "specialist competences". Key aspects of allergy management include patient education, trigger identification and avoidance, prevention measures, medications for long-term disease control and relief of acute exacerbations as well as patient desensitization and allergen specific immunotherapy. In contrast to prior recommendations such as those shown in Fig. 2, our recommendations for specialist competences in Fig. 3 provide a more general view of the skills and knowledge required of the allergy specialist. Alongside our recommendations, the allergy specialist will likely fulfil various roles: 1) medical expert, 2) communicator, 3) collaborator, 4) leader, 5) health advocate, 6) scholar, 7) professional, as described within the CanMEDS framework developed by the Royal College of Physicians and Surgeons of Canada.^26^ We recognize that the specific roles undertaken by the allergy specialist and associated competences are dependent on facilities and training available in each region. In light of rapid developments in diagnostic and treatment techniques, we have left the specifics unstipulated. In all cases, a physician who orders specialist allergy tests must also have the competence to interpret the results or have access to an allergy specialist with requisite competence to interpret them, to avoid untargeted excessive testing and inappropriate management recommendations. This has become an important issue with the advent of component resolved diagnostics which should only be ordered by those with the knowledge and competence to understand the significance of the results. We also wish to re-emphasize the consideration of psychosocial factors in allergic disease as a "core competence" expected of all physicians, due to the significance this holds in the patient experience.

## Specialty training heterogeneity

There are several factors which may inhibit international standardization of competences. Our proposed "specialist competences" (Fig. 3) are designed for the role of the allergy specialist. However, we recognize the complexity of this recommendation in view of the diversity of allergy specialty training and practice worldwide.

Allergy is not yet universally recognized as a specialty. In a recent European survey, 14 out of 36 responding countries reported the recognition of an allergology specialty only, 9 countries reported a subspecialty only, and a further 9 countries reported both.^10^ Allergology was not recognized as either a specialty or subspecialty^10^ in 5 countries. To overcome the specialty training heterogeneity in Europe, the Union of European Medical Specialists (UEMS) in collaboration with EAACI established training requirements for the specialty in the 27 EU member states.^27^ A similar survey, conducted across Latin America in 2013, found that only 56% of countries recognized allergology as either a specialty or subspecialty.^28^ Isolated reports from other parts of the world corroborate a mixed picture; in Hong Kong, for example, allergology is recognized as a full specialty,^29^ while in South Africa^30^ and Israel, it is recognized as a subspecialty only.^31^ Alongside the heterogeneity of specialty recognition, there is an absolute lack of allergy specialists worldwide. In Europe, there is one allergy specialist for every 50,000,^22^ in the United States, 1 for every 75,000^22^ and in Hong Kong, 1 for every 1.46 million^29^ inhabitants, respectively. This further complicates the standardization and harmonization of allergy care.

While the allergy burden is increasing worldwide,^2^ its prevalence is most commonly reported in Westernized countries;^2^ thus, it is logical for Australasia, Europe, and North America to have recognition of an allergology specialty. Allergology specialization is most effective in the context of a tertiary referral system, in which patients with complex allergies are referred to a central hub of specialist-led multidisciplinary team (MDT) allergy services, known in some countries as an “allergy center”. Due to variable distribution of population and infrastructure design, this system is not the norm.^29^

Furthermore, in areas where the allergy specialty is recognized, there is significant variation between allergy specialty training programs.

For example, in the United States the Allergy Immunology programs are accredited by the Accreditation Council for Graduate Medical Education (ACGME), in which the applicant first completes a three-year residency in either Internal Medicine or Pediatrics followed by 2 years of additional training in Allergy and Immunology. Thus, 5 years of postgraduate training are required to become eligible to sit for the American Board of Allergy and Immunology (ABAI).^32^ The Brazilian Association of Allergy and Immunopathology (ASBAI) similarly requires a two-year residency in Allergy and Clinical Immunology in addition to previous training in Internal Medicine.^33^ Meanwhile, in Europe, allergology specific training can range from 1 to 5 years, with Finland requiring the most extensive total training time of over 7 years.^10^ The specifics of clinical exposure can also vary, with some countries such as South Africa recommending a laboratory placement is included as part of training, to encourage research progression alongside clinical practice.^30^

Considering this heterogeneity, further work is needed to evaluate the similarities and differences between allergy specialty training pathways and specialist roles internationally. This will aid evaluation of competence recommendations, to ensure they are suitable for global implementation and adaptation according to local infrastructure. This will require the continuing engagement of international bodies of allergy specialists to harmonize and standardize specialty training programs. Health experts in the countries lacking allergy as a specialty should encourage local authorities to set-up specialist programs in allergy and clinical immunology to cover unmet needs.

## Multidisciplinary awareness and skills in allergy education and training

Although it is crucial that highly complex and risky procedures in the allergy field, such as desensitization to drugs, food, and/or aeroallergens should be performed in the specialized clinic by well-trained experts, it is crucial that physicians are aware of the multidisciplinary facets of allergy care, including patient education, psychological care, and dietetic interventions. In some areas, these interventions are fulfilled by the specialist or subspecialist physician, whilst in other settings these services are provided by specialist allied health professionals. We understand that there is variation in how these tasks are distributed within the MDT according to local infrastructures. However, it is important to ensure these competences are well-understood so that if input from the specific allied health professional is not available, such tasks can be fulfilled by the physician, nurse, or medical assistant. The distribution of competences within the allergy MDT as part of integrated care pathways is discussed further in an accompanying paper, “Harmonizing allergy care - integrated care pathways and multidisciplinary approaches”, which discusses roles of other members of the allergy MDT such as nurses and pharmacists and how competences can be cascaded through integrated care pathways. In this paper, we emphasize the importance of the following areas: patient education, psychology, and dietetics.

### Patient education and training

Developing a mutually agreed management plan among the treating physician, allied health professionals, the patient and their caregivers helps to cascade effective allergy care into the patient's home and local settings. If patients and their families lack adequate information and signposting from their physician, they may seek support from unverified sources on the internet such as discussion forums, which a recent analysis shows may encourage non-evidence based and potentially dangerous practices such as using alternative medicine treatments or testing for allergy at home.^34^

Non-adherence has a serious impact on patient health and outcomes, as medication can only be effective if taken correctly and consistently as prescribed. There are numerous patient-related factors that contribute to non-adherence: poor treatment-related knowledge, psychological factors such as anxiety and depression, worries about specific side effects, and developmental factors in the transition from childhood to adolescent and adult care.^35^ Similar factors are related to the non-carriage and/or reluctance to use an epinephrine auto-injector.^36^ Achieving an accord between the physician and the patient, including training the patients and their caregivers in coping with their specific care needs, is more likely to result in better concordance with the agreed management plan. It is vital that physicians and the wider healthcare team learn to work closely with their patients to develop these plans together as equal partners and to address any underlying concerns.

### Psychology

The large psychosocial impact of allergic disease calls for a more prominent role for applied psychology within allergy education and training. Allergic diseases can significantly affect quality of life, with limitation of activities and negative effects on self-image. Food allergy is associated with a considerable quality of life burden, as self-management involves constant vigilance to avoid coming into contact with food allergens.^37^ This affects social life, such as eating out or staying over with friends, school life, including an increased risk of bullying, and familial relationships. The evidence shows that responding to both psychological and physical needs can improve patient outcomes and reduce overall treatment costs.^38^^,^^39^

With this in mind, we include the patient perspective and psychosocial aspects of allergy as minimum components in the undergraduate curricula. This would facilitate the practice of psychosocial care as a "core competence" in allergy practice (Fig. 3), so that physician care is psychologically informed, in line with the boundaries of their role. Physicians should also be aware of when to involve psychologists in the care of the patient where availability allows, who can dedicate additional targeted therapies to maximize the patient's physical and mental wellbeing, particularly crucial in the transition process from pediatric to adult care.^40^

### Dietetics

It is vital that physicians have an awareness of the role of dietetics in allergy, including early introduction of food allergens for allergy prevention,^41^ taking an allergy-focused diet history,^42^ and managing food allergies across the spectrum of IgE^43^ and non-IgE mediated intolerances.^44^^,^^45^ This dietetic care is ideally directed by a specialist dietitian where availability permits. The role of the allergy dietitian in tolerance induction has also been highlighted.^46^ An allergy-focused diet history can help to both identify the allergen and determine nutritional status,^42^ which informs decision-making regarding the most appropriate management plan and frequency of follow-up.^47^ Dietetic involvement is crucial in the preparation and organization of food challenges^48^ as well as in the progressive reintroduction of food-stuffs.^49^

With this in mind, a recent EAACI task force report has promoted recognition of allergy specialist dieticians as part of MDT care, particularly with respect to the long-term care of pediatric cases for whom both allergy remission or progression are possible.^47^ The authors also suggest that common understanding of dietetic competences by other healthcare professionals can aid prompt, appropriate referral to allergy specialist dieticians. Accordingly, we emphasize that recognition of the need for MDT working is a "core competence", expected of all physicians (Fig. 3). Furthermore, for allergy specialists, a high-level understanding of the role of dietetics should be presumed as a crucial element of formulating patient-centered management.

## Ethical standards in the allergy world

Following on from our recommendations, we draw attention to some ethical challenges faced by all physicians but contextualized to allergy training and practice.

The ability to critically appraise research is important in the era of evidence-based medicine, as physicians seek to self-educate and keep their knowledge up to date. Skills include the identification of conflicts of interest, publication and political bias, and an understanding of techniques such as “ghost-writing” where a company pays a key opinion leader to put their name to an article written by a professional health-writer to subtly promote an agenda, promote a product or inculcate a bias in the reader.^50^^,^^51^ It is important that physicians can explain the principles of evidence-based medicine to patients, particularly in light of fraudulent claims, such as RK Chandra's reports of reduced allergy outcomes in babies fed a partially hydrolyzed whey hydrolysate formula.^52^^,^^53^

We also promote awareness of the unethical practices of over-investigation and the use of unvalidated, unstandardized, “alternative”, “unorthodox” allergy diagnostic testing and treatment modalities.^54^ Patients who are unable to access specialist help may be more likely to turn to these unvalidated tests, particularly those which have become commercially available. The results from such tests may persuade parents to overly restrict a child's diet, potentially resulting in malnutrition and permanent effects on growth and development.^55^ Ensuring patients and their families are aware of these questionable practices enables intelligent autonomy and may encourage them to support other families, promoting the spread of reliable knowledge around the patient community.

Ethical challenges can also be found in the application of postgraduate training in allergy. Continued medical education and continued professional development are hallmarks of best practice and necessary for all graduates to stay up to date with the evidence base of their specialty. In allergy, this is facilitated by numerous postgraduate courses, which may be viewed by graduates as an efficient means of gaining competences, especially if their undergraduate education lacked allergy content. However, these courses vary greatly in their depth of content, standards of assessment and methods of accreditation. Some courses may convey additional prestige by their affiliation with well-known institutions. The current heterogeneity leads to inappropriate flexible interpretation of the graduate's new “expertise” which may lead them to practice above their true level of skills and knowledge. Depending on local infrastructure, this has the potential to create false reputations of expertise, facilitate unethical financial gain and, most importantly, jeopardize patient care. It is important that the registration and assessment of postgraduate training is made transparent to ensure that participants can safely practice within their claimed competence. There have been local attempts to standardize postgraduate training, such as through the development of Entrustable Professional Activities (EPAs): observable modules of competence-based work supervised by a senior clinician.^56^ These may be a helpful tool to assess the outcomes of training opportunities more widely, alongside the competence recommendations made in Fig. 3 and taking into account variability in practice between regions. Further work is needed to create international guidance for the standardization and correct application of postgraduate training opportunities.

## Conclusion

Allergy is a rapidly developing specialty, which remains unrecognized in some geographical locations. There are significant deficiencies in allergy training at the undergraduate and postgraduate level in many parts of the world with poor standardization of allergy specialist training worldwide. With the rapid progress in understanding of mechanisms and management of allergic diseases, a growing number of diagnostic and management options have become available. A holistic, multidisciplinary approach is necessary for the provision of high-quality services, from primary care to specialist settings. This requires understanding of communicating effectively with the patient, as well as timely implementation of psychological and dietetic interventions. A unified approach to allergy education and training worldwide with harmonization of undergraduate education has the potential to significantly improve knowledge, skills, and practice and improve the quality of patient care.

## Abbreviations

World Allergy Organization (WAO), International Collaboration in Allergy, Asthma, and Immunology (iCAALL), European Academy of Allergy and Clinical Immunology (EAACI), Multidisciplinary Team (MDT), Union of European Medical Specialists (UEMS), Entrustable Professional Activities (EPA).

## Ethics statement

The authors declare that no experiments were performed on humans or animals for this article.

## Confidentiality of data

The authors declare that no patient data appear in this article.

## Agreement to publish the work

All authors consent to the publication of this work.

## Author contributions

ML, JOW, TG, BM, SGD, LD and SB made contributions to the conception and design.

CV, DM, LD, SB, AK assisted with literature searching.

LD and SB authored the first draft of the manuscript.

CV, TG, JG, SGD, ML, JOW, LD, SB, BM, HH, RVG, CDQ, RGA and ES provided critical contributions to the manuscript. CV, ML, LD and SB contributed to the graphic works.

DM provided supervision of the manuscript.

All authors critically reviewed and approved the final version of the submitted manuscript.

## Financial support

None to declare.

## Declaration of competing interest

None of the authors have any conflicts of interest to report related to this work.
